# O.4.3-8 Associations of subjective social status with physical activity and sedentary time among lower secondary school students

**DOI:** 10.1093/eurpub/ckad133.194

**Published:** 2023-09-11

**Authors:** Katja Rajala

**Affiliations:** Jamk University of Applied Sciences, Likes, Finland; katja.rajala@jamk.fi

## Abstract

**Purpose:**

The low level of physical activity among young people has become a common societal concern. This study sought new information on the background to adolescents’ physical activity. The aim was to determine whether adolescents’ subjective social status is related to their physical activity.

**Methods:**

The data was collected as part of follow-up research and evaluation of the Finnish Schools on the Move programme in 2012–2015. The data was collected through student surveys, measuring adolescents’ physical activity with accelerometers, and observing and interviewing secondary school students. The associations between adolescents’ subjective social status and physical activity, as well as the factors related to the subjective social status were studied using both quantitative and qualitative methods.

**Results:**

Adolescents’ subjective social status was associated with physical activity. The subjective social status in school was positively associated with whole-day moderate- to vigorous-intensity physical activity. Adolescents’ high perceived social status in school was significantly associated with physical activity among boys and girls during break times, participation in planning break-time activities among girls, and participation in arranging common school events among boys and girls. Subjective social status in school was negatively associated with sedentary time in school. A high subjective social status in school was also associated with more opportunities for activity created with schoolmates. A high subjective social status was also associated with a wider environment for physical activities in school, which were related to the interactive relationships between adolescents. There were school premises that not everyone had access to, and one limiting factor may be the low subjective social status in school.

**Conclusions:**

The different subjective experiences of school-based social networks are linked to adolescents’ physical activity and should be taken into account when considering the possibilities of increasing adolescents’ physical activity. Although the foundation of an active lifestyle is largely built on one’s own family, the school still has the potential to compensate for differences in family background with its own actions. These interventions should place greater emphasis on peer relations and youth participation rather than the role of school staff.

**Support/Funding Source:**

The Ministry of Education and Culture of Finland.

